# Doubly protected ester prodrug of 5-aminolevulinic acid for enhanced cell uptake and photoinactivation^†^

**DOI:** 10.21203/rs.3.rs-9033698/v1

**Published:** 2026-04-09

**Authors:** Hailey S. Sanders, Karolina A. Rooney, Marissa A. Panethiere, Kirk M. Atkinson, Bradley D. Smith

**Affiliations:** Department of Chemistry and Biochemistry, University of Notre Dame, Notre Dame, Indiana 46556, USA

**Keywords:** 5-aminolevulinic acid, photodynamic therapy, protoporphyrin IX, esterase, lipophilic

## Abstract

Photodynamic therapy (PDT) employs a molecular photosensitizer combined with light to induce localized cellular toxicity. The natural product, 5-aminolevulinic acid (5-ALA), is used in clinical PDT as a precursor drug molecule that is converted inside cancer cells to the photosensitizer, protoporphyrin IX (PpIX). However, the therapeutic efficacy of 5-ALA is limited by its relatively short chemical lifetime under physiological conditions and its poor bioavailability. To obviate these limitations, we have developed 5-eMAL as a doubly protected ester prodrug of 5-ALA with greater chemical stability and improved capacity to enter cells. Intracellular esterase activity converts 5-eMAL into free 5-ALA for subsequent biosynthesis into PpIX. Cell culture experiments with three different cancer cell lines (HepG2, 4T1, A459) showed greatly enhanced production of intracellular PpIX when the cells were treated with 5-eMAL compared to cells treated with 5-ALA. Inhibition of the membrane peptide transporters in HepG2 cells did not lower PpIX formation when the cells were treated with 5-eMAL suggesting that cellular uptake of 5-eMAL is independent of the membrane transporters. Cell photoinactivation experiments using blue light irradiation produced much greater cell death when the cells were pre-treated with 5-eMAL compared to cells that were pretreated with 5-ALA. Cell microscopy showed that the photoinactivated cells were co-stained by the fluorescent indicators Annexin V FITC and Propidium Iodide suggesting non-apoptotic cell death. Cell studies using different nanoparticle formulations of 5-eMAL also produced high levels of intracellular PpIX highlighting the potential for in-vivo application.

## Graphical Abstract

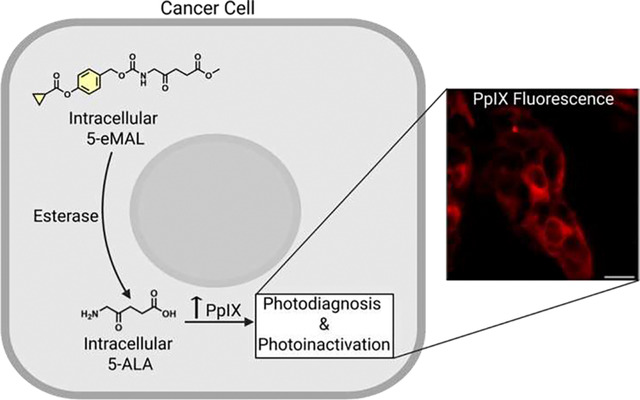

## 1. Introduction

5-aminolevulinic acid (5-ALA) is a naturally occurring non-proteinogenic amino acid and the starting material for intracellular heme biosynthesis.[1] In cancer cells, the activity of ferrochelatase, the enzyme that converts the penultimate compound protoporphyrin IX (PpIX) into heme, is often low leading to temporary accumulation of PpIX which is a fluorescent molecule and efficient oxygen photosensitizer.[2] 5-ALA is used clinically for photodynamic therapy (PDT) of actinic keratoses and other skin conditions,[2] [3] and it is also approved for fluorescence guided surgery of gliomas.[4] [5] [6] A combination of multiple factors determines the level of accumulated PpIX in cancer cells that have been treated with 5-ALA. [7] A necessary requirement is efficient cellular uptake of the dosed 5-ALA which is a polar amino acid, that primarily enters cells through membrane peptide transporters.[8] [9] [10] A related physical drawback with polar 5-ALA is its limited penetration through skin and tissue. To overcome these bioavailability problems researchers have pursued different medicinal chemistry strategies such as structurally modified prodrugs, chemical additives that enhance cell permeability, and nanoscale drug delivery systems. [10] [11] [12] [13]

Focusing on 5-ALA prodrugs, many chemical derivatives have been reported over the last few decades, and evaluated for a range of clinical improvements such as enhanced storage stability, increased skin permeation, and increased accumulation of PpIX inside cancer cells. [9] [11] [14] [15] Many carboxylic ester derivatives of 5-ALA have been tested and two of them are clinically approved, namely, the methyl ester for topical treatment of actinic keratosis and basal cell carcinoma and the hexyl ester for blue-light cystoscopy detection of bladder cancer. [16] There are far fewer reports of amine derivatives of 5-ALA primarily because the amide or carbamate derivatives of 5-ALA are relatively stable and less likely to converted by intracellular enzymes into 5-ALA for subsequent bioconversion to PpIX.[17] [11]

**Scheme 1:**
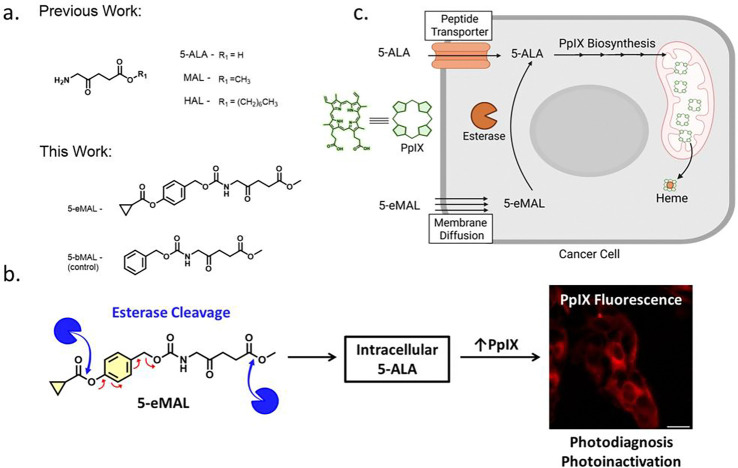
(a) Chemical structures of 5-ALA and derivatives used in this study. (b) Esterase based conversion of 5-eMAL into intracellular 5-ALA triggering protoporphyrin IX (PpIX) accumulation for cancer photodiagnosis and photoinactivation. (c) Cartoon depicting cell entry of 5-ALA or 5-eMAL and bioconversion to intracellular PpIX.

In recent years, there has been impressive development of new classes of pharmaceutical protecting groups with self-immolative linkers that can be triggered by a range of biochemical processes to undergo fragmentation and release payload. [18] Some of this prodrug technology has been used to make amine-modified 5-ALA derivatives. Examples include doubly protected 5-ALA derivatives with a carboxylic ester group that must be cleaved by intracellular esterase activity (Scheme 1a), and an amine-linked carbamate group that must be fragmented by a self-immolative cascade triggered by a disulfide cleavage reaction, [15] [19] [20] enzyme-promoted glucoronate hydrolysis, [21] or enzyme-promoted hydrolysis of a phosphate group. [14] [22] [23] In each of these cases, the two ends of the doubly protected 5-ALA derivative must be cleaved by two separate and independent biochemical processes. We reasoned that a pharmaceutical advantage might be gained by developing a doubly protected 5-ALA prodrug that can be converted to 5-ALA by a single enzyme with the capacity to remove protecting groups from both ends of the same prodrug molecule. Here, we realize this vision by reporting 5-eMAL as the first example of a doubly protected prodrug of 5-ALA that can be cleaved at both ends by esterase activity to generate 5-ALA (Scheme 1). We show that cultured cancer cells treated with 5-eMAL produce much higher levels of intracellular PpIX than cells treated with 5-ALA or 5-ALA prodrugs that only have the carboxylic acid protected as an ester group. Additionally, cell photoinactivation experiments produced much greater cell death when the cells were pre-treated with low doses of 5-eMAL compared to cells that were pre-treated with equivalents doses of 5-ALA or 5-ALA mono-ester prodrug.

## 2. Experiments

### 2.1. Reagents and Instruments

Reagents and solvents were purchased from Sigma-Aldrich, VWR, Oakwood, Thermo Fisher, Ambeed or TCI and used without further purification unless stated otherwise. Column chromatography was performed using Biotage Sfär columns or using silica gel (230–400 mesh). ^1^H and NMR spectra were recorded on a Bruker 400 NMR spectrometer. Chemical shifts are presented in ppm and referenced by residual solvent peak. High-resolution mass spectrometry (HRMS) was performed using a time-of-flight (TOF) analyzer with electrospray ionization (ESI). Compound **1** was prepared by a previously reported method.[24] Graphs were made using GraphPad Prism (version 10.4.2).

### 2.2. Synthesis

#### 2.2.1. MAL

5-aminolevulinic acid hydrochloride (665 mg, 3.897 mmol) was dissolved in 200 mL of MeOH and heated to 60 °C. Concentrated HCl (3 drops) was added, and the reaction was stirred at 60 °C for 24 hours. Rotary evaporation provided MAL as a tan solid (541 mg, 94% yield) that was sufficiently pure for subsequent use. ^1^H NMR (400 MHz, MeOD) δ 4.02 (d, J = 1.1 Hz, 2H), 3.66 (s, 3H), 2.83 (t, J = 6.3 Hz, 2H), 2.68 (t, J = 6.0 Hz, 2H). HRMS (ESI+): calculated for C_6_H_11_NO_3_ [M + H]^+^ 146.0812, found 146.0810.

#### 2.2.2. HAL

5-aminolevulinic acid hydrochloride (635 mg, 3.79 mmol) was dissolved in 10 mL of 1-heptanol containing 3 drops of concentrated HCl. The reaction was heated to 66° for 20 hours and then cooled to room temperature. Rotary evaporation provided HAL as a pale-orange solid (930.2 mg, 92%) that was sufficiently pure for subsequent use. ^1^H NMR (400 MHz, DMSO-d_6_) δ 8.35 (s, 3H), 3.99 (t, J = 6.7 Hz, 2H), 3.94 (d, J = 4.9 Hz, 2H), 2.79 (t, J = 6.5 Hz, 2H), 2.53 (t, J = 6.5 Hz, 2H), 1.59–1.50 (m, 2H), 1.31–1.22 (m, 8H), 0.85 (t, J = 6.5 Hz, 3H). HRMS (ESI+): calculated for C_12_H_24_NO_3_ [M + H+]^+^ 230.1751, found 230.1759.

#### 2.2.3. 5-eMAL

Compound **1**[24] (500.0 mg, 2.6 mmol, 1 eq), was dissolved in triethylamine (725 μL, 5.2 mmol, 2 eq), acetonitrile (3 mL) and dimethylformamide (1 mL) and stirred at room temperature for 10 minutes. N,N disuccinimidyl chloride (1.0 g, 3.90 mmol, 1.5 eq) was added and stirred at room temperature for an hour. Next, the reaction mixture was cooled to 0 °C, MAL (755.2 mg, 5.2 mmol, 2 eq) and triethylamine amine (725 μL, 5.2 mmol, 2 eq) were added and the reaction stirred at to 0 °C for one hour. Next, the temperature was allowed to reach room temperature and the reaction mixture stirred for another 2 hours. Finally, dichloromethane (20 mL) was added, and the organic phase was washed with dilute HCl (20 mL 0.01 M HCl), and the crude product was obtained after rotary evaporation. The crude product was purified using column chromatography (silica gel, 0–70% EtOAc in hexanes) followed by recrystallization in methyl tert-butyl ether to afford a white solid (480 mg, 51%) ^1^HNMR (400 MHz, CDCl3) δ 7.36 (d, J = 8.3 Hz, 2H), 7.08 (d, J = 8.2 Hz, 2H), 5.46 (s, 1H), 5.09 (s, 2H), 4.14 (d, J = 5.0 Hz, 2H), 2.75 – 2.69 (m, 2H), 2.68 – 2.63 (m, 2H), 1.83 (tt, J = 8.0, 4.6 Hz, 1H), 1.16 (dt, J = 8.3, 5.0 Hz, 2H), 1.02 (dq, J = 8.2, 4.4, 3.4 Hz, 2H). HRMS (ESI+): calculated for C_18_H_21_NO_7_ [M + H]^+^ 386.1210, found 386.1207.

#### 2.2.4. 5-bMAL

A solution of MAL (125 mg, 0.69 mmol, 1 eq), 2 mL of dry DMF and 2 mL of dry MeCN was chilled to 0°C, afterwards triethylamine (288 μL, 2.07 mmol, 3 eq) was added. Benzyl chloroformate (148 μL, 1.03 mmol, 1.5 eq) was added and stirred letting reach room temperature for 2 hours. The reaction was evaporated, and the crude was dissolved in 15 mL of DCM and washed with 50 mL of brine, dried with MgSO4 and purified by column chromatography (SiO2, 0–60% EtOAc in Hexanes) to afford 5-bMAL as a clear oil (55.3 mg, 29% yield). 1H NMR (400 MHz, Chloroform-d) δ 7.36 – 7.31 (m, 5H), 5.50 (s, 1H), 5.11 (s, 2H), 4.14 (d, J = 4.9 Hz, 2H), 3.67 (s, 3H), 2.74 – 2.69 (m, 2H), 2.65 (t, J = 5.9 Hz, 2H). HRMS (ESI+): calculated for C_14_H_18_NO_5_ [M + H]^+^ 280.1179, found 280.1171.

### 2.3. Cell Culture Conditions

HepG2 (HB-8065 ^™^) human liver hepatocellular carcinoma cells were purchased from ATCC ^®^ and maintained in EMEM Media supplemented with 10% fetal bovine serum (FBS), and 1% penicillin-streptomycin. 4T1 (CRL-2539 ^™^) mouse mammary cancer cells were purchased from ATCC ^®^ and maintained in RPMI-1640 Media supplemented with 10% FBS, and 1% penicillin-streptomycin. A549 (ATCC^®^ CCL-185^™^) human lung adenocarcinoma cells were purchased from ATCC ^®^ and maintained in F-12K Media supplemented with 10% FBS, and 1% penicillin-streptomycin. In each case, the culture was maintained at 37 °C, 5% CO_2_.

### 2.4. Protoporphyrin IX (PpIX) Extraction from Cells

HepG2 cells (6.0 × 10^3^) were seeded in a 96-well dish and grown 48 hours in supplemented EMEM. A549 cells (1 × 10^4^) were seeded in a 96-well dish and grown 24 hours in supplemented F-12K. 4T1 cells (1 × 10^4^) were seeded in a 96-well dish and grown 24 hours in supplemented RPMI-1640. Stock solutions (10 mM) of the ALA derivatives, 5-ALA, 5-eMAL, 5-bMAL, MAL, or HAL, were made in a 1:1 mixture of DMSO and phosphate buffered saline (PBS). The ALA derivatives were diluted into FBS-Free DMEM and added onto cells. For PpIX concentration study, 0 – 50 μM of ALA derivatives were added. For transporter inhibition, 100 μM 5-ALA and 50 μM 5-eMAL were. Once ALA derivatives solutions were added onto the cells, the cells were incubated for three hours. After incubation with ALA, the media containing ALA derivative was removed and 200 μL HCl (2 M) was added onto the cells and the cells then incubated 30 minutes to extract PpIX. The HCl solution was diluted into 1X PBS (1:5) and PpIX fluorescence spectra measured on the Horiba Fluoromax-4 spectrometer. ^‡^

### 2.5. Cell Microscopy

HepG2 cells (5.0 × 10^4^) were seeded onto a 4-well Ibidi Treat 35 mm glass bottom dish and grown 24 hours. Media was removed and 5-eMAL or 5-ALA (5 μM) in FBS-Free DMEM was added and the cells incubated for three hours. The media was replaced with Opti-MEM and fluorescence microscopy was conducted using a Zeiss Axiovert 100 TV epifluorescence microscope (63x oil immersion) equipped with an X-Cite 120Q light source with metal-halide lamp. PpIX fluorescence micrographs were acquired using a custom blue excitation, red emission filter (Ex: 387/11 nm, dichromatic mirror: 409 nm, Em: 655/40 nm). For each micrograph, 6 separate fields were selected and analyzed for PpIX fluorescence intensity using ImageJ software. A background subtraction with a rolling ball radius of 100 pixels was applied and the average mean pixel intensity (MPI) of the entire field was determined. Microscopy experiments were repeated two times, and the averages were plotted with p values calculated using OriginPro. ***p < 0.01.

### 2.6. Cell Peptide Transporter Inhibition Studies

Stock solutions (10 mM) of gamma-aminobutyric acid (GABA) and 4-(aminomethyl)benzoic acid (PAMBA) were made in sterile 1X PBS. HepG2 cells were seeded (6.0 × 10^3^) in a 96-well dish and grown for 72 hours in EMEM. The EMEM was aspirated and aliquots of 5-eMAL (50 μM) or 5-ALA (100 μM) in combination with either GABA or PAMBA (100 μM) were added onto cells and the cells incubated for three hours. The ALA and peptide inhibitor solution was removed, the PpIX extracted and its fluorescence measured as described above.

### 2.7. Photoinactivation of Cells

HepG2 cells (1.0 × 10^4^) were seeded in a 96-well plate and grown 48 hours in EMEM. Media was replaced with 5-eMAL or 5-ALA (0 – 10 μM) in FBS-Free DMEM and the cells incubated for 3 hours. The external media was removed and the cells washed once with 1X PBS. The PBS was replaced with Opti-MEM, then irradiated for ten minutes at 37 °C with 405 nm light from an LED array located directly under the 96-well plate (LEDA-x, Amuza Inc) and set to 6 mW output. The Opti-MEM was replaced with EMEM and the cells allowed to recover overnight. The cell metabolic activity in each microwell was measured using a standard MTT assay. The EMEM was replaced with a solution 3-(4,5-dimethylthiazol-2-yl)-2,5-diphenyltetrazolium bromide (MTT; 0.5 mg/mL) in EMEM and the cells incubated for 4 hours. A solution of sodium dodecyl sulfate (0.1 g/mL) containing HCl (20 μM) was added to cells, and the absorbance (570 nm) was recorded for each well. IC_50_ values were determined by using GraphPad Prism (version 10.4.2) with non-linear curve fitting and the study was replicated.

### 2.8. Fluorescence Microscopy of Photoinactivated Cells

HepG2 cells (5.0 × 10^4^) were seeded onto a 35 mm glass bottom dish (MatTek) and grown over 48 hours. Media was replaced with 5-eMAL (5 μM) in FBS-Free DMEM and the cells incubated for three hours. The external media was replaced with Opti-MEM, and the cells irradiated for ten minutes at 37 °C with 405 nm light from an LED array (6 mW output) located directly under the 96-well plate (LEDA-x, Amuza Inc). The Opti-MEM was removed and the cells stained with Annexin V-FITC and Propidium Iodide (Sigma Aldrich) following the manufacturer’s protocol. That is, 1X Binding Buffer containing Annexin-V-FITC (5 μL of stock solution per 1 mL Binding Buffer) and Propidium Iodide (PI, 10 μL of stock solution per 1 mL Binding Buffer) were added to the cells followed by a 10 minute incubation. The external solution was removed, the cells washed with 1X PBS buffer, then Opti-MEM was added for fluorescence microscopy using a Zeiss Axiovert 100 TV epifluorescence microscope (63x oil immersion) equipped with an X-Cite 120Q light source with metal-halide lamp. The micrographs were acquired using FITC channel (Ex: 485/20 nm, Em: 524/24 nm) for imaging Annexin V-FITC and TxRed channel (Ex: 562/40 nm, Em: 624/40 nm) for imaging Propidium Iodide.

### 2.9. Cell Treatment Using Nanoparticle Formulations of 5-eMAL

#### 2.9.1. POPC Liposome Formulation

A thin film of 90% (molar percentage) 1-palmitoyl-2-oleoyl-sn-glycero-3-phosphocholine (POPC) and 10% 5-eMAL was created by evaporating a mixed chloroform solution in a glass test tube and drying the film overnight under high vacuum. The lipid film was rehydrated using a solution of 1X PBS and briefly vortexed for 15 seconds followed by 15 freeze-thaw cycles where one cycle consisted of 1.5 minute bath in liquid nitrogen followed by 1.5 minute bath in warm water (50 °C) The resulting liposome stock solution had a total polar lipid concentration of 1 mM with 10% 5-eMAL. HepG2 cells (1.0 × 10^4^) were seeded in a 96-well dish and grown 48 hours in supplemented EMEM. The EMEM was removed and FBS-Free DMEM containing 5-eMAL. POPC liposomes were added onto cells so that the final concentration of 5-eMAL was 10 μM. The cells were incubated for three hours, the external solution replaced with 200 μL of HCl (2 M), and the cells incubated for 30 minutes. The HCl solution was diluted into 800 μL 1X PBS in a quartz cuvette and the PpIX fluorescence intensity was recorded.

#### 2.9.2. Solid Lipid-Polymer Nanoparticle Preparation

A rapid nanoprecipitation method was used to prepare 5-eMAL-encapsulated nanoparticles. An organic phase was created by mixing 10 mg of poly(lactic-co-glycolic acid) (PLGA), 3 mg of 1,2-distearoyl-sn-glycero-3-phosphoethanolamine-poly(ethylene glycol) (DSPE-PEG), 7 mg of lecithin, and 2 mg of cholesterol in 1 mL of 99% ethanol to give a final ratio of 1:0.3:0.7:0.2. Complete dissolution was achieved after rapid stirring at 40°C for 10 minutes. After cooling to room temperature, 5-eMAL was added to achieve a 2.0% (w/w) solution. A separate aqueous phase was prepared by dissolving 10 mg of BSA in 2 mL of deionized water to create a 0.5% w/v solution. Nanoprecipitation was induced by rapidly injecting 1 mL of the organic phase into 2 mL of the aqueous phase (acting as the stabilizer) with vigorous stirring. The mixture was stirred at room temperature for an additional 20 minutes and sonicated using a pulse sonicator (frequency - 20 kHz, power - 130 W, CV18 converter and (1/8”) 3 mm probe, Sonics and Material Inc.) with short pulses of 30 seconds for 5 minutes to facilitate the formation of 5-eMAL encapsulated lipid-polymer nanoparticles. The resulting mixture was then diluted with 2 mL of HEPES buffer (100 mM, pH 7.4) to reduce the ethanol content and the nanoparticles centrifuged at 4000 rpm for 3 minutes using an Amicon Ultra-4 centrifugal filter [MWCO 10 kDa]. The washing process was repeated for an additional two rounds to remove excess ethanol and unencapsulated 5-eMAL. After the washing steps, the nanoparticle dispersion was filtered through a 0.45 μm filter and stored at 4°C. HepG2 cells (1.0 × 10^4^) were seeded in a 96-well dish and grown 48 hours in supplemented EMEM. The EMEM was removed and FBS-Free DMEM containing the 5-eMAL nanoparticles was added onto cells so that the final concentration of 5-eMAL was 10 μM. The cells were incubated for three hours, the external solution replaced with 200 μL of HCl (2M), and the cells incubated for 30 minutes. The HCl solution was diluted into 800 μL 1X PBS in a quartz cuvette and the PpIX fluorescence intensity was recorded.

#### 2.9.3. Pluronic F-127 Micelle Formulation

A stock solution of Pluronic F-127 (15% w/v) was prepared in 1X PBS and allowed to sit overnight at 4° C. A stock solution of 5-eMAL (10 mM) was prepared using the Pluronic F-127 (15% w/v) solution. HepG2 cells (5.0 × 10^3^) were seeded in a 96-well dish and grown for 48 hours in supplemented EMEM. The EMEM was removed and FBS-Free DMEM containing 5-eMAL in Pluronic F-127 solution was added onto cells so that the final concentration of 5-eMAL was 10 μM. The cells were incubated for three hours, the external solution replaced with 200 μL of HCl (2M), and the cells incubated for 30 minutes. The HCl solution was diluted into 800 μL 1X PBS in a quartz cuvette and the PpIX fluorescence intensity was recorded.

## 3. Results and Discussion

### 3.1. Prodrug Synthesis and Stability

**Scheme 2:**
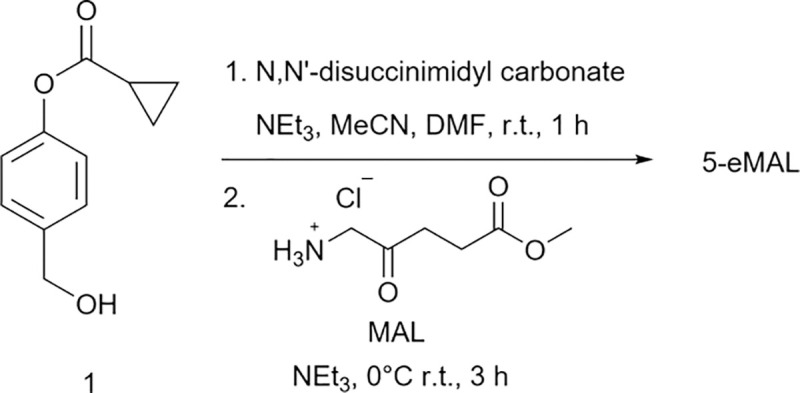
Synthesis of 5-eMAL.

The control compounds MAL, Hal, and 5-bMAL were prepared using straightforward synthetic methods. In addition, the known compound **1** [24] was converted into a mixed carbonate and reacted with MAL to give 5-eMAL in 51% yield (Scheme 2). A solution of 5-eMAL was monitored by ^1^H NMR over a 24 hour period and there was no spectral change indicating high chemical stability (Scheme S1) which is in contrast to the known propensity of 5-ALA and 5-ALA carboxylic esters to undergo dimerization reactions under neutral conditions to produce biologically inactive products. [11] [25] The higher chemical stability of 5-eMAL is due to chemical conversion of the nucleophilic amine within 5-ALA to a less reactive carbamate group. We have previously shown that a cyclopropyl ester group is a good substrate for many esterases, [24] and we reasoned that hydrolysis of the cyclopropyl ester in 5-eMAL would trigger a self-immolative fragmentation pathway that liberated that protected amine group (see Scheme S2).

### 3.2. Intracellular PpIX Formation

**Fig. 1:**
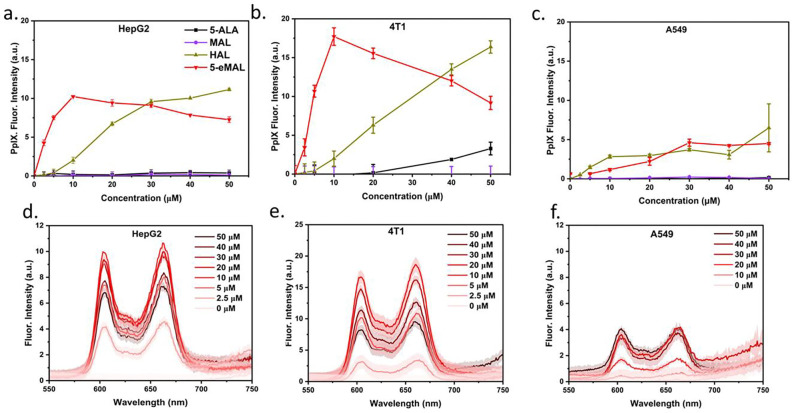
(a-c) Protoporphyrin IX (PpIX) fluorescent intensity measured from (a) HepG2, (b) 4T1, or (c) A549 cells treated three hours with 5-ALA, 5-eMAL, MAL, or HAL (0 – 50 μM). Bars represent standard error (N = 3). (d-f) Fluorescent spectra of PpIX extracted from (d) HepG2, (e) 4T1, or (f) A549 cells treated three hours with 5-eMAL (0 – 50 μM). Shaded region represents error (N = 3).

Addition of 5-ALA to cultured cells is known to increase the intracellular biosynthesis of PpIX. The high polarity of 5-ALA limits cell entry by plasma membrane diffusion and there is strong evidence that cellular uptake of externally dosed 5-ALA occurs primarily via cell membrane peptide transporters.[8] We expected uncharged and lipophilic 5-eMAL to rapidly enter cells by direct diffusion through the plasma membrane and that intracellular esterases would hydrolyze the ester groups at both ends of the molecule, producing high levels of intracellular 5-ALA for subsequent biosynthesis into PpIX whose red fluorescence can be quantified. This hypothesis was tested by conducting cell experiments that incubated separate batches of cultured cancer cells with incrementally varied doses of 5-ALA, 5-eMAL, and two control compounds, 5-ALA methyl ester (MAL) and 5-ALA heptyl ester (HAL). In each case, the amount of intracellular PpIX that was produced after each incubation was quantified using a standard fluorescence spectroscopy assay that measures the amount of PpIX extracted from the cells using strong acid. ^‡^ Three cell-lines were studied, namely, hepatocellular carcinoma cells (HepG2), mouse mammary cancer (4T1), and lung adenocarcinoma (A549). As shown in Fig. 1a, negligible PpIX fluorescence was observed in HepG2 cells that were treated with doses of 5-ALA or MAL up to 50 μM. This is an agreement with previous work showing that several hundred micromolar of 5-ALA is needed to produce significant levels of intracellular PpIX fluorescence. [11] [26] In contrast, HepG2 cell treatment with 5-eMAL at concentrations as low as 2.5 μM generated strong PpIX fluorescence, with a near constant signal produced when the dose was above 10 μM. We observed that a 5-eMAL dose of 20 μM or more induced the HepG2 cells to detach from the inner surface of the dish, suggesting that the high level of biosynthesized PpIX was causing cell death. The experiments that treated HepG2 cells with HAL also produced a measurable increase in fluorescence, but a dose of 30 μM was required to attain the same signal intensity gained by adding 10 μM 5-eMAL. A similar trend was obtained for the experiments using 4T1 cells (Fig. 1b) where a 10 μM dose of 5-eMAL produced a much higher level of PpIX fluorescence than the other compounds. In A549 cells the addition of 5-eMAL and HAL produced similarly low levels of PpIX fluorescence but still much higher fluorescence than treatment with 5-ALA or MAL (Fig. 1c). Together, these results demonstrate that cell treatment with 5-eMAL produces much higher levels of intracellular PpIX compared to 5-ALA or MAL, and it is also significantly more effective than HAL in two of the three cell-lines that were tested (i.e., HepG2 and 4T1 cells). The lower PpIX levels observed when 5-eMAL or HAL is added to A549 cells is attributed to PpIX efflux by the ABC transporter G2 (ABCG2) that is elevated in this cell-line.[27]

### 3.3. Cell Microscopy

**Fig. 2:**
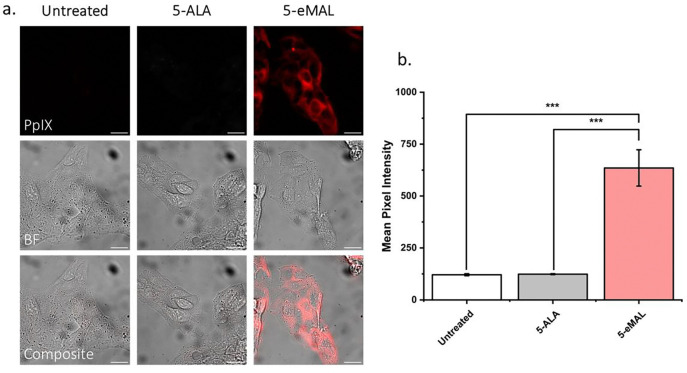
(a) Representative micrographs of HepG2 cells left untreated, incubated three hours with 5-ALA (5 μM), or incubated three hours with 5-eMAL (5 μM). (b) Average mean pixel intensity of HepG2 cells. Bars represent standard error of a single microscopy experiment where N = 6 micrographs. *** p<0.001. Ex: 387/11 nm; Dichromatic mirror: 409 nm; Em: 655/40 nm. Scale bar = 30 μm.

When excited by blue light excitation, PpIX emits red fluorescence that can be detected for photodiagnostic imaging.[28] Confirmation that a low dose of 5-eMAL can produce observable PpIX fluorescence in living cells was gained by conducting fluorescence microscopy experiments using HepG2 cells and a custom filter set to detect PpIX emission (Ex: 387/11 nm; Em: 655/40 nm). Shown in Fig. 2a are representative micrographs of untreated HepG2 cells or cells incubated for three hours with either 5-ALA or 5-eMAL (5 μM) prior to imaging. As expected, there was negligible red fluorescence in the untreated cells or the cells incubated with 5-ALA. In contrast, the cells treated with 5-eMAL produced strong red fluorescence. For each condition, 6 micrographs were acquired (Fig. S1 – Fig. S3) and the digital images were analyzed to give the quantitative comparison of mean pixel intensity (MPI) in Fig. 2b. The microscopy study was repeated using living 4T1 cells, and the same trend was observed, i.e., incubation with 5 μM 5-eMAL produced strong intracellular fluorescence while the same concentration of 5-ALA produced no detectable signal (Fig. S4 – Fig. S7).

#### 3.3.1 5-bMAL Control Study

**Fig. 3:**
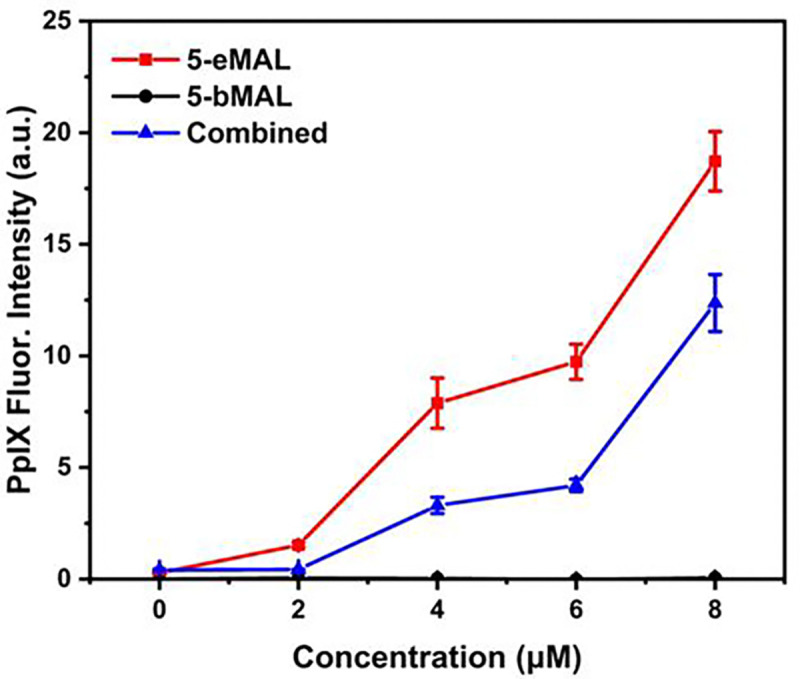
Fluorescent intensity of PpIX extracted from HepG2 cells treated with 5-eMAL, control 5-bMAL, or a 1:1 combination added to cells (0 – 8 μM) for three hours at 37 °C. Bars represent error (N = 3).

A chemical requirement for converting 5-eMAL into 5-ALA is hydrolysis of the cyclopropyl ester bond in 5-eMAL followed by self-immolative fragmentation to liberate the free amine group. This requirement was confirmed by conducting cell incubation experiments with the control derivative, 5-bMAL, whose structure has the amine nitrogen protected as a relatively unreactive benzyl carbamate group. HepG2 cells were incubated for three hours with 5-eMAL, 5-bMAL, or a 1:1 mixture of both derivatives, and in each case the resulting intracellular PpIX was quantified by cell extraction and fluorescence spectroscopy (Fig. 3). As described above, cells treated with incremental amounts of 5-eMAL produced relatively high and dose-dependent levels of PpIX. In contrast, no detectable PpIX fluorescence was observed when the cells were treated with 5-bMAL, suggesting that the carbamate linkage was not cleaved by intracellular enzymes. Cells treated with a 1:1 mixture of both derivatives produced PpIX levels that were commensurate with the 5-eMAL dose, suggesting that the 5-bMAL does not inhibit the intracellular enzyme(s) that hydrolyze 5-eMAL. Overall, hydrolysis of the cyclopropyl ester is a requirement for intracellular conversion of 5-eMAL into 5-ALA and ultimately PpIX.

### 3.4. Peptide Transporter Inhibition

**Fig. 4:**
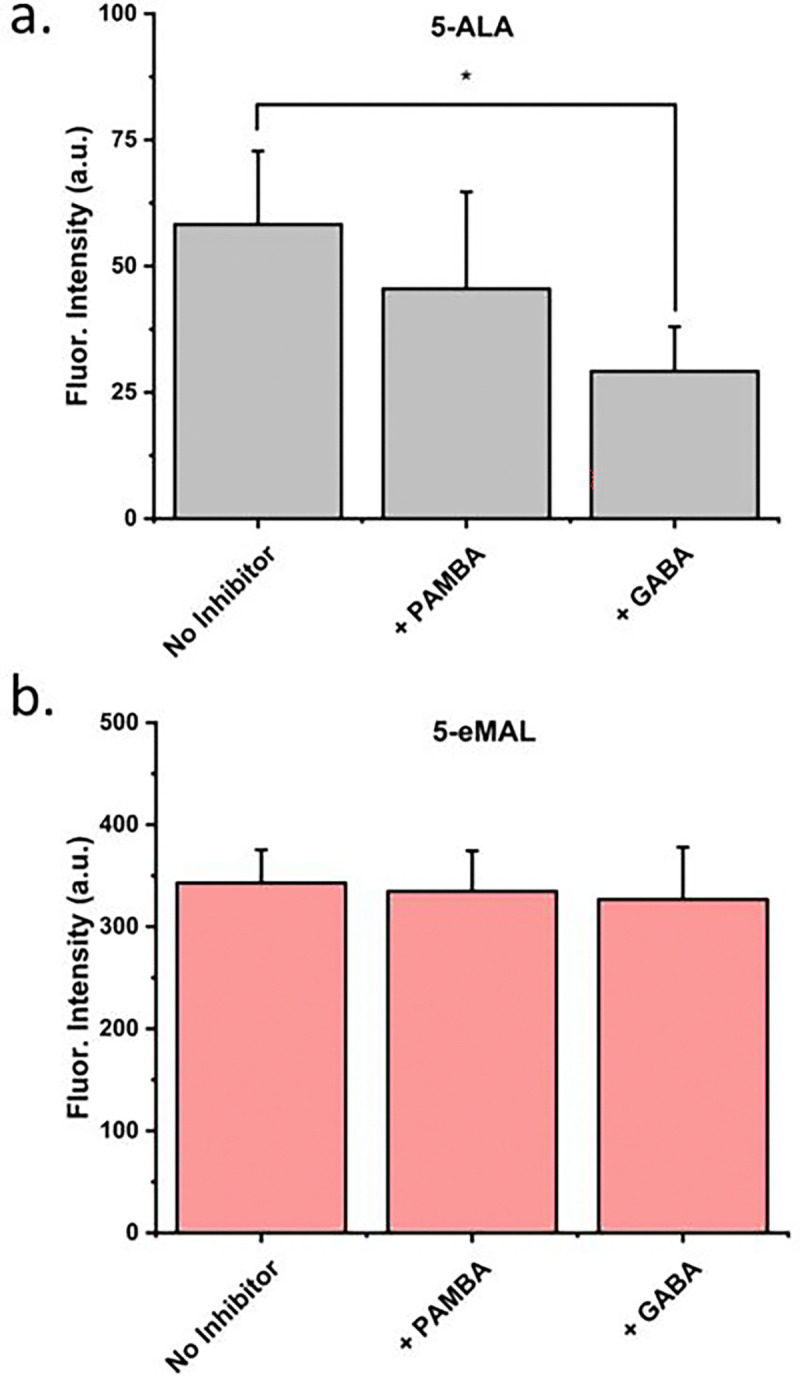
Fluorescence intensity of PpIX extracted from HepG2 cells that had been treated for three hours with a binary mixture of (a) 5-ALA (100 μM) and 100 μM PAMBA or GABA, or (b) 5-eMAL (50 μM) and 100 μM PAMBA or GABA. Bars represent error (N = 3). * p < 0.05.

There is good evidence that cell uptake of exogenous 5-ALA is facilitated by endogenous membrane peptide transporters. [2] [8] Previous work has demonstrated that cell uptake of 5-ALA is inhibited by pre-treatment of the cells with small molecule inhibitors of the membrane peptide transporters. For example, studies have shown that γ-aminobutyric acid (GABA) and p-aminomethylbenzoic acid (PAMBA) are effective inhibitors of 5-ALA entry into human adenocarcinoma cells. [29] [30] [31] The charge neutrality and lipophilicity of 5-eMAL suggests that it can directly enter cells by passive diffusion and thus can bypass the endogenous membrane peptide transporters. This hypothesis was tested by conducting a series of transporter inhibition studies that determined if the presence of GABA or PAMBA lowered the amount of intracellular PpIX that was produced when cells were dosed with 5-ALA or 5-eMAL. The experiments incubated HepG2 cells for three hours with 5-ALA (100 μM) mixed with PAMBA or GABA (100 μM), or 5-eMAL (50 μM) mixed with PAMBA or GABA (100 μM). In each case the amount intracellular PpIX was quantified by cell extraction and fluorescence spectroscopy. As shown by the graph in Fig. 4a, the presence of peptide transport inhibitors (GABA or PAMBA) significantly reduced the amount of intracellular PpIX for cells treated with 5-ALA. In contrast, the presence of the peptide transport inhibitors did not alter the amount of intracellular PpIX for cells treated with 5-eMAL (Fig. 4b), indicating that 5-eMAL does not enter cells via membrane peptide transporters.

### 3.5. 5-eMAL Photoinactivation of Cells

**Fig. 5:**
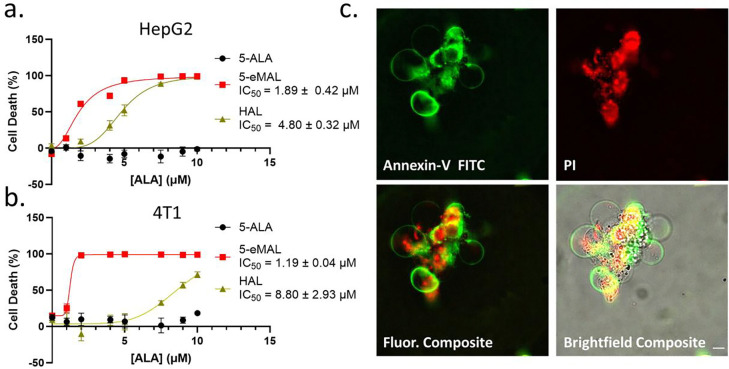
Cell death curves for: (a) HepG2 cells, or (b) 4T1 cells, incubated for three hours with 5-ALA, 5-eMAL, or HAL (0 – 10 μM) and then irradiated with 405 nm (6 mW) light for 10 minutes. Data is representative of one study and error bars represent standard deviation of three separate measurements. (c) Fluorescent micrographs showing HepG2 cells that had been incubated for three hours with 5-eMAL (5 μM) and then irradiated with 405 nm light (10 minutes). The irradiated cells were stained with Annexin-V FITC (Ex: 485/20 nm, Em: 524/24 nm) and Propidium Iodide (PI) (Ex: 562/40, Em 624/40). Scale bar = 30 μm.

The intracellular PpIX that is produced by treatment with 5-ALA is typically irradiated with blue light to generate cytotoxic reactive oxygen species (ROS). [32] [33] We reasoned that an increased level of intracellular PpIX should produce a higher amount of photogenerated ROS and greater amount of cell death. This proposition was tested by conducting HepG2 and 4T1 cell photoinactivation studies that compared the phototoxic effects of 5-ALA, HAL, and 5-eMAL at different dosing concentrations up to 10 μM. (Fig. 5). Before the photoinactivation studies commenced we conducted a set of cell metabolic activity assays using HepG2 cells to show that the three compounds did not exhibit significant dark toxicity at the doses used for photoinactivation (Figure S8). The cell photoinactivation studies incubated in separate batches of HepG2 or 4T1 cells for 3 hours with incrementally increased doses of 5-ALA 5-eMAL, and HAL (up to 10 μM). The cells were maintained in a 96-well plate that was irradiated for ten minutes at 37 °C with 405 nm light from an LED array located directly under the 96-well plate. The cell metabolic activity was measured for the different conditions. The entire cell photoinactivation study was conducted three times and the data was compiled to give a set of cell death curves. Inspection of the curves in Fig 5 shows a large difference in photoinactivation using the three different compounds. Blue light irradiation of cells treated with > 2 μM 5-eMAL produced a large decrease in cell metabolic activity. In comparison, a significantly higher dose of HAL was needed to produce the same phototoxicity effect and 5-ALA produced no detectable phototoxicity at the tested concentrations. The photoinactivation IC_50_ values for 5-eMAL and HAL were determined to be 1.8 μM and 4.8 μM in HepG2 cells, and 1.2 μM and 8.8 μM in 4T1 cells. In absence of light, there was no loss in metabolic activity for cells treated for 3 hours with 5-ALA, 5-eMAL, or HAL (Figure S9 and S10).

Microscopic characterization of the cell death following photoinactivation using 5-eMAL was performed by staining the photoinactivated cells with the fluorescent markers, Annexin V FITC and Propidium Iodide (PI). Annexin V FITC stains the membranes of dead and dying cells, while PI will only enter porous necrotic cells and stain the internal DNA. [34] The representative cell micrographs in Fig. 5c show strong staining by both fluorescent markers and also reveal the appearance of prominent membrane bubbles; both microscopic features suggest a non-apoptotic cell death pathway.

### 3.6. Nanoparticle Formulation of 5-eMAL

**Fig. 6:**
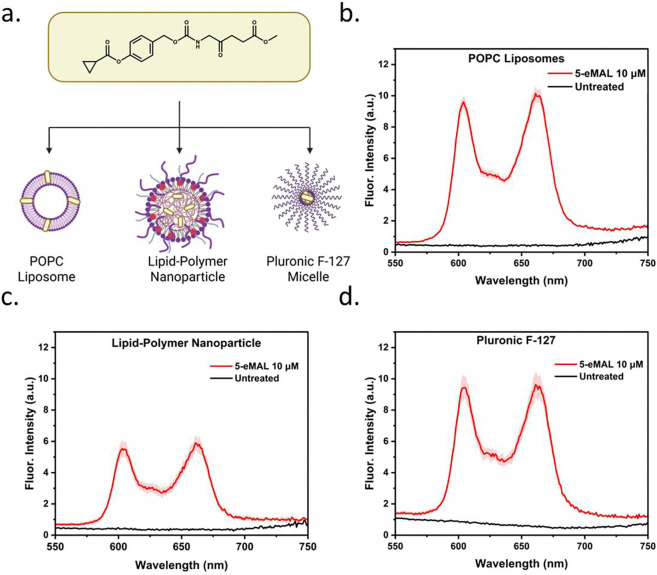
(a) Cartoon depicting POPC liposomes, solid lipid/polymer nanoparticles, or pluronic F-127 micelles, each loaded with 5-eMAL. Fluorescence spectra of PpIX extracted from HepG2 cells after incubation for three hours with the three different nanoparticle formulations (final concentration of 5-eMAL = 10 μM). Shaded regions represent error (N = 3).

The use of nanoparticles to package and deliver 5-ALA or 5-ALA prodrugs for in-vivo applications has several attractive features such as protection of the compounds from preliminary biodegradation, capacity to coat the nanoparticle surface with disease targeting units, and capacity to encapsulation other drugs or diagnostic agents within the same nanoparticle for enhanced performance. The preclinical research literature includes reports of nanoparticles containing polar 5-ALA in aqueous sites [35] [36] or lipophilic 5-ALA esters held in hydrophobic regions. [37] [38] [39] To evaluate the feasibility of 5-eMAL for future in-vivo imaging and phototherapy studies we prepared three separate nanoparticle formulations and tested them for capacity to deliver 5-eMAL into cultured cells and produce biosynthesized PpIX. As summarized in Fig. 6a, the three nanoparticle formulations were (a) liposomes composed of POPC, [40] (b) solid lipid-polymer nanoparticles composed of PLGA, DSPE-PEG, lecithin, and cholesterol, [41] and (c) micelles composed of pluronic F-127. [42] Standard methods were used to produce the nanoparticles containing 5-eMAL, and the formulations were added to cultured Hep2G cells. After a three hour incubation the PpIX was extracted and quantified using fluorescence spectroscopy. The results in Fig. 6b–d demonstrate that in each case there was substantial amount of PpIX produced supporting the feasibility of these nanoparticle 5-eMAL formulations for future in-vivo imaging and phototherapy studies.

## 4. Conclusions

5-ALA is a polar molecule and primarily enters cells via membrane peptide transporters to subsequently produce biosynthesized PpIX. We have prepared a new 5-ALA prodrug called 5-eMAL, whose structure contains ester groups at both ends of the molecule. Studies with several different cancer lines (Hep2G, 4T1, A549) showed that cell treatment with low concentrations of 5-eMAL (< 10 μm) produced high levels of intracellular PpIX. Under the same conditions a 50 μM dose 5-ALA produced negligible PpIX. Indeed, low doses of 5-eMAL produced more intracellular PpIX than equivalent doses of the mono-ester 5-ALA prodrugs, MAL and HAL. Cell entry by 5-eMAL is not reduced by the presence of membrane peptide transport inhibitors, indicating that 5-eMAL diffuses through the cell plasma membrane and is subsequently cleaved by intracellular esterases to produce 5-ALA which feeds into the heme biosynthesis pathway. The potential of 5-eMAL to be cleaved at both ends of the structure by the same esterase activity differentiates it from the other “doubly protected” 5-ALA prodrugs in the literature that must be cleaved at each end by distinctly different chemical processes. The higher production of intracellular PpIX led to increased levels of cell photoinactivation, and cells treated with 5-eMAL produced photoinactivation than cells treated with equivalent doses of 5-ALA or the mono-ester prodrug HAL. Fluorescence microscopy indicted that the photoinactivation process produced non-apoptotic cell death. Successful formulation of 5-eMAL within several different nanoparticle suggests a feasible delivery strategy for future in-vivo applications.[43] In this regard, selective cancer targeting should occur in cells that overexpress the intracellular esterase enzymes that rapidly convert 5-eMAL into 5-ALA, or by using nanoparticles coated with cancer targeting units that efficiently deliver the 5-eMAL to cancerous tissue for subsequent transfer of the 5-eMAL into the cancer cells. Looking beyond cancer PDT it is worth noting that 5-ALA-PpIX PDT has shown promise for treatment of other diseases such as bacterial infection and burn-wound healing.[44] [45] In addition, 5-ALA treatment alone (without light irradiation of the biosynthesized PpIX) has therapeutic potential to manage various metabolic disorders.[46] All of these new and different pharmaceutical applications are likely to benefit from improved methods of 5-ALA drug delivery such as the 5-eMAL prodrug system described here.

## Supplementary Material

Supplementary data to this article can be found online at https://doi.org/######

This is a list of supplementary files associated with this preprint. Click to download.

• SandersPPSESI.pdf

## Data Availability

The data that support the findings of this study are available from the corresponding author upon a reasonable request.

## Footnotes

‡ The PpIX extraction assay produces two emission peaks (see Figure 1) with the lower-wavelength emission attributed to protonated PpIX. [47–49] The peak intensity ratios did not change as the experiments progressed, so the assays tracked the intensity of the longer-wavelength peak as a measurement of relative intracellular PpIX concentration.
